# Identification of Novel miRNAs and Their Target Genes in the Response to Abscisic Acid in *Arabidopsis*

**DOI:** 10.3390/ijms22137153

**Published:** 2021-07-01

**Authors:** Syed Muhammad Muntazir Mehdi, Sivakumar Krishnamoorthy, Michal Wojciech Szczesniak, Agnieszka Ludwików

**Affiliations:** 1 Laboratory of Biotechnology, Institute of Molecular Biology and Biotechnology, Faculty of Biology, Adam Mickiewicz University in Poznan, Uniwersytetu Poznanskiego 6, 61-614 Poznan, Poland; syemeh@amu.edu.pl (S.M.M.M.); sivkri@amu.edu.pl (S.K.); 2 Institute of Human Biology and Evolution, Faculty of Biology, Adam Mickiewicz University in Poznan, Uniwersytetu Poznanskiego 6, 61-614 Poznan, Poland; michal.szczesniak@amu.edu.pl

**Keywords:** miRNA, abscisic acid, ABI1 PP2C, MAPKKK17, MAPKKK18, *Arabidopsis thaliana*

## Abstract

miRNAs are involved in various biological processes, including adaptive responses to abiotic stress. To understand the role of miRNAs in the response to ABA, ABA-responsive miRNAs were identified by small RNA sequencing in wild-type *Arabidopsis*, as well as in *abi1td*, *mkkk17*, and *mkkk18* mutants. We identified 10 novel miRNAs in WT after ABA treatment, while in *abi1td*, *mkkk17*, and *mkkk18* mutants, three, seven, and nine known miRNAs, respectively, were differentially expressed after ABA treatment. One novel miRNA (miRn-8) was differentially expressed in the *mkkk17* mutant. Potential target genes of the miRNA panel were identified using psRNATarget. Sequencing results were validated by quantitative RT-PCR of several known and novel miRNAs in all genotypes. Of the predicted targets of novel miRNAs, seven target genes of six novel miRNAs were further validated by 5′ RLM-RACE. Gene ontology analyses showed the potential target genes of ABA-responsive known and novel miRNAs to be involved in diverse cellular processes in plants, including development and stomatal movement. These outcomes suggest that a number of the identified miRNAs have crucial roles in plant responses to environmental stress, as well as in plant development, and might have common regulatory roles in the core ABA signaling pathway.

## 1. Introduction

Over the last few decades, there has been intensive research into the physiological effects of abscisic acid (ABA) [1] on plants. From sprouting to senescence, ABA regulation is a feature of almost the whole life cycle of plant growth and development. Thus, ABA controls organ and root growth, and it modulates metabolism and a host of other processes in plants [2]. ABA is also critically involved in plant responses to abiotic stress, for example, by altering root architecture and changing patterns of growth and quiescence. ABA controls plant responses via numerous effector proteins, but the most important route for the regulation of plant growth and development is the core ABA signaling pathway. This linear pathway comprises three classes of protein: the ABA receptors themselves, variously named pyrabactin resistance/pyrabactin resistance-like/regulatory component of the ABA receptor (encoded by the *PYR*/*PYL*/*RCAR* genes); the negative regulators of the pathway, the protein phosphatase 2C (*PP2C*) group A family; and the positive regulators *SNF1*-related protein kinases type 2 (SnRK2 genes). Other kinases such as MAP kinases function downstream of the ABA core pathway: MAPKKK17 and MAPKKK18 are regulated by the ABA core signaling module, and these activate C group MAPKs via MKK3; these downstream kinase modules probably contribute to the generation of a robust, long-term signal that maintains ABA-dependent responses under chronic stress conditions [3]. MAPKKK18 directly interacts with ABI1 protein phosphatase, a negative regulator of ABA signaling, which comprises the core signaling module of ABA and is regulated by the ubiquitin-proteasome pathway [4]. Transcription of *MAPKKK17* and *MAPKKK18* is induced by ABA, and these genes seem to have redundant functions in ABA signaling [5]. Thus, phosphorylation cascades are a fundamental component of ABA signaling [6]. However, it has also been shown that ABA can affect the expression levels of several microRNAs (miRNAs), which regulate downstream effector genes [7].

miRNAs are ~21-nucleotide (nt)-long, single-stranded small RNAs that play important monitoring roles in eukaryotes. miRNA genes code for long pri-miRNA (primary miRNA) transcripts with imperfect stem-loop secondary structures, which are transcribed by RNA polymerase II in plants [8,9]. These transcripts are processed into ~70 nt pre-miRNAs and subsequently duplexes of miRNA/miRNA* by *DCL-1* (dicer-like enzyme 1) in association with a dsRNA-binding protein, HYL-1. These duplexes are methylated by a dsRNA methylase, HEN1 (*HUA ENHANCER 1*), and loaded into *AGO1* (Argonaute1) [10,11,12], and then, with the help of an exportin homolog protein called *HASTY* [13,14], transported to the cytoplasm, where they are cleaved into ~22 nt mature miRNAs. Mature miRNA strands form part of a multiprotein complex, the RNA-induced silencing complex (*RISC*), where they guide the cleavage of matching target mRNAs by *AGO1*, which possesses RNA-binding domains such as *PAZ* and *PIWI* [14,15]. The effect of miRNAs on complementary target mRNAs is to promote their cleavage post-transcriptionally or to interfere with their translation [16]. Stresses and plant hormones such as auxin and ABA often regulate the expression of miRNAs, which are important regulators of development and stress responses in plants [17,18,19]. Identification of novel miRNAs and their target mRNAs and elucidation of the cellular context in which they function remain important for understanding the role of miRNAs in gene regulation.

Deep sequencing technology has led to the identification of numerous miRNAs in plant genomes from species such as Italian sweet pepper [20], tea, mulberry, Chinese cabbage, turnip, longan (*Dimocarpus longan* Lour.), bread wheat, *Artemisia annua*, *Populus euphratica*, *Moringa oleifera*, and *Solanum lycopersicum* [21,22,23,24,25,26,27,28,29,30]. The following are some examples of these miRNAs, many of which are involved in specific signaling pathways. One of the most abundant and evolutionarily conserved miRNAs in plants, miR156, accumulates in seedlings to high levels, which then decrease with age [31,32,33]. miR160 and miR165/166 contribute to the embryogenic potential of *Arabidopsis* somatic tissues by regulation of the LEC2-controlled pathway of auxin biosynthesis [34]. A loss-of-function approach suggested that miR159a/miR159b predominantly regulates *MYB33* and *MYB65* [35], while ABA induction of miR159 also showed that this miRNA controls the transcript levels of the *MYB* factors, *MYB33* and *MYB101* [18]. After three-hour exposure to 10 μM ABA, pre-miR846 was suppressed (with up to 60% reduction in levels) and, even after the withdrawal of exogenous ABA, pre-miR846 levels continued to decrease over a 24 h period [36]. However, the question of whether ABA is involved in core signaling and MAPKKK pathways via miRNAs remains to be clarified in *Arabidopsis*.

In the current study, we aimed to identify novel ABA-responsive miRNAs and their putative target mRNAs and to investigate whether and how the ABA response in Arabidopsis involves miRNA regulation of the ABA signaling pathway.

## 2. Results

### 2.1. Small RNA Sequencing of *Arabidopsis* WT Col-0 and abi1td, mkkk17, and mkkk18 Mutants after ABA Treatment

Molecular and genetic studies have revealed that in response to environmental stress, complex signaling pathways are induced in plants, leading to a host of diverse responses [37]. The stress hormone abscisic acid is known to mediate plant responses to environmental stress provoked by drought, salinity, cold, heat, and ozone, among others [38,39]. In particular, the core ABA signaling pathway is important for regulation of the stress response. MiRNAs mediated in the ABA core signaling pathway are largely unknown. Therefore, to gain insight into the role of miRNAs in the ABA core pathway, we performed small RNA profiling of the *ABI1*, *MKKK17*, and *MKKK18* knockout lines and compared them to the wild-type Col-0 control plants. The MAPKKK17/MAPKKK18 cascade is activated by the ABA core module. Moreover, MAPKKK18 activity is regulated by the ABA core element ABI1 PP2C. The MAPKKK18 and a close paralogue kinase, MAPKKK17, seem to exert partially redundant functions in the ABA signaling network [3,4]. Therefore, to identify known and novel ABA-responsive miRNAs, triplicate samples of *A. thaliana* WT Col-0, *abi1td*, *mkkk17*, and *mkkk18* seedlings were treated with 100 µM ABA solution for 4 h alongside untreated controls. Total RNA was then prepared as described in the Materials and Methods section and sent to Macrogen Inc. (Seoul, South Korea) for small RNA library construction and sequencing on an Illumina HiSeq 2500 system. Each of the 24 individual libraries produced an average of more than 10 million reads. Details of the miRNA statistics are given in Table 1.

**Figure 1:**
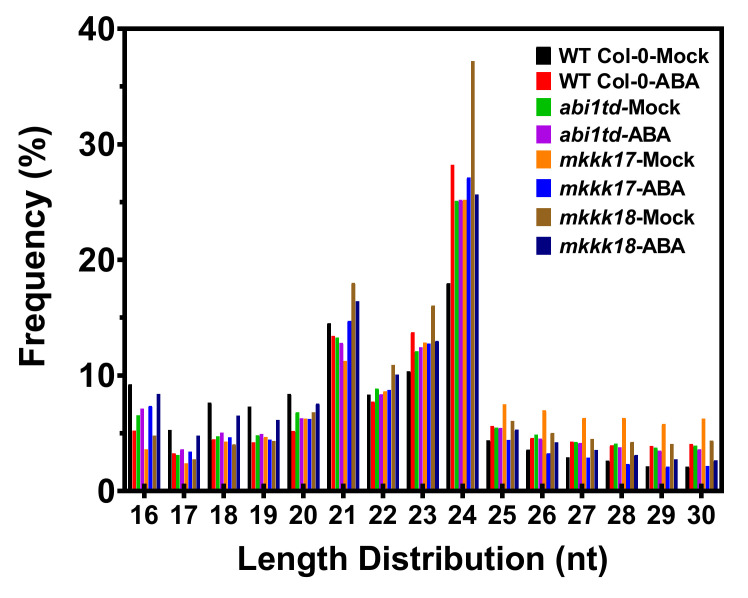
Length distribution of small RNAs. Length distribution of small RNAs in samples of *Arabidopsis* WT Col-0, *abi1td*, *mkkk17*, and *mkkk18* mutants before or after four hours of 100 µM ABA treatment (mock- and 100 µM ABA-treated). The X-axis represents the small RNA length (nucleotides) and the Y-axis represents the percentage of small RNA reads.

Overall, the miRNAs ranged from 16 to 30 nt in size across all libraries of all genotypes (Figure 1). Different size categories of these RNAs are associated with different functions. Thus, 24 nt sRNAs mostly induce gene silencing by heterochromatin maintenance or RNA-dependent DNA methylation of target genes, while post-transcriptional gene silencing is generally controlled by 21 nt sRNAs [40,41,42]. The length distribution of small RNAs changed after ABA treatment, showing an increased or decreased number of 18, 19, 21, 22, 23, and 24 nt small RNAs. Interestingly, the 21 and 24 nt-sized groups were the most predominant categories of miRNA across all genotypes and for both mock- and ABA-induced samples in our dataset (Figure 1; Supplementary Table S2). These results suggest that the expression profiles of small RNAs are altered by ABA in all genotypes and demonstrate the presence of a complex and varied sRNA population in *Arabidopsis*.

### 2.2. Identification of Known and Novel miRNAs

**Figure 2:**
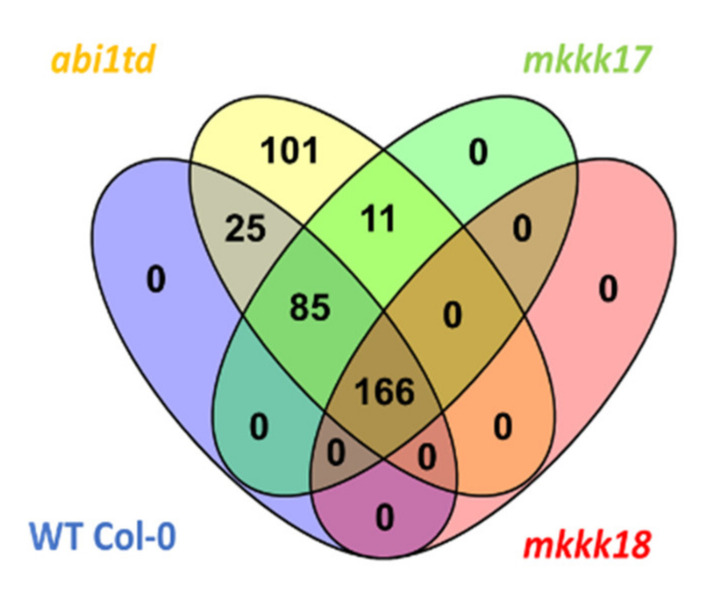
Venn diagram showing the common and specific total small RNAs identified expressed in all samples of ABA-treated vs. mock-treated *Arabidopsis* WT Col-0, *abi1td*, *mkkk17*, and *mkkk18* mutants.

We aligned all miRNAs identified in the various libraries against known miRNA datasets in miRBase release 22. Then, all identified miRNAs were matched against the *Arabidopsis* genome downloaded from the TAIR database. After these screening steps, we identified 225, 315, 212, and 148 known miRNAs and 51, 73, 50, and 18 novel miRNAs in the WT Col-0, *abi1td*, *mkkk17*, and *mkkk18* libraries, respectively (Figure 2 and Supplementary Tables S3–S6).

**Figure 3:**
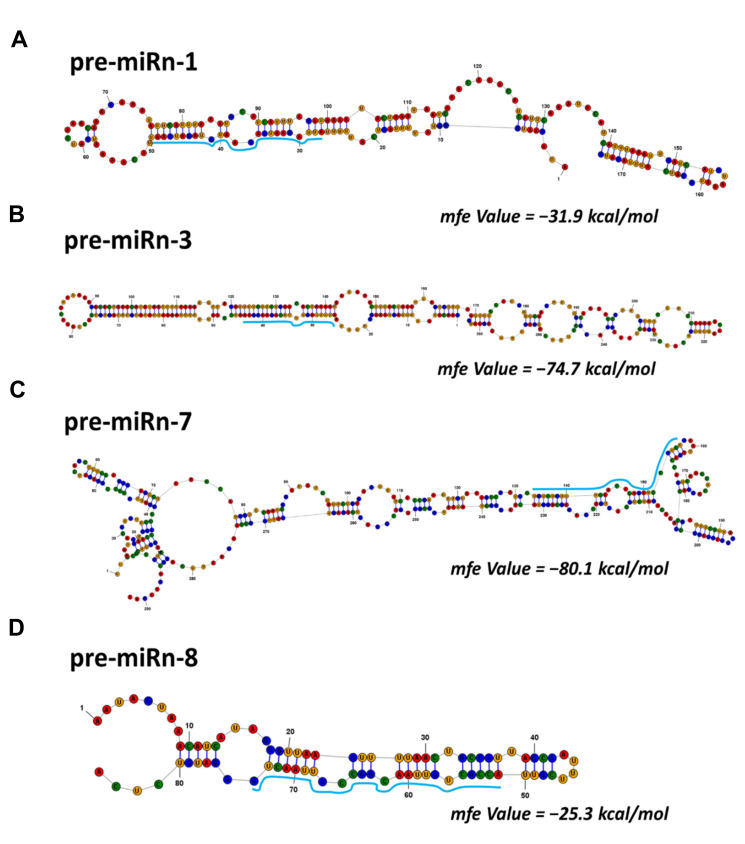
Secondary structures for the novel miRNA precursors. Predicted hairpin secondary structures for the potential novel miRNA precursors of **(A)** miRn-1 (**B**) miRn-3, (**C**) miRn-7, and (**D**) miRn-8. Nucleotide bases of mature miRNA are highlighted in light blue. The actual size of each putative precursor might differ slightly from its shown length since it was not identified experimentally. The computed minimum free energy (MFE) of the thermodynamic ensemble is reported. RNAstructure version 6.2 software was employed to evaluate the stem-loop structure with default parameter settings.

Normalized reads (reads per million, RPM) were used to evaluate the expression levels of each miRNA (Supplementary Table S7). Table 2 shows the top fifteen most highly expressed miRNAs based on the number of clean reads for all genotypes and treatments, together with their fold change values. The *Arabidopsis* genome was used as a reference to search for the potential precursors of novel miRNA candidates (Supplementary Table S8), and the putative precursor sequences were examined for the potential to form secondary structures using RNAstructure v. 6.2 software (Mathews group, Rochester, NY, USA). Representative stem-loop secondary structures for several novel pre-miRNAs are shown in Figure 3A–D and Supplementary Figure S2A–C.

**Figure 4:**
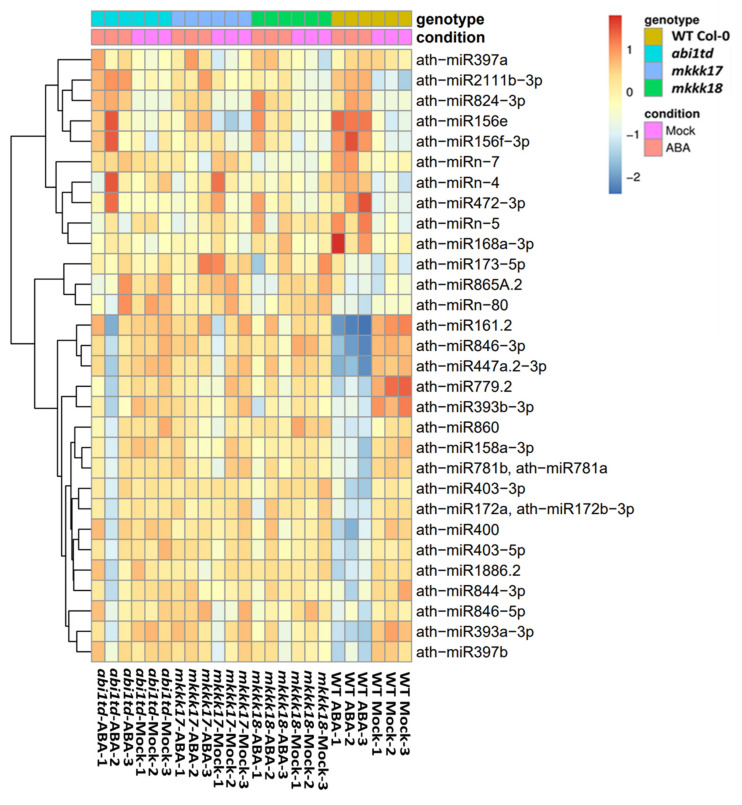
Distribution and expression pattern of known and novel miRNAs in all libraries. The hierarchical clustering analysis by heatmap of ABA-responsive miRNAs from samples of ABA-treated vs. mock-treated *Arabidopsis* WT Col-0, *abi1td*, *mkkk17*, and *mkkk18* mutants. Heatmap showing hierarchical clustering of 30 differently expressed miRNAs are displayed by distance and complete cluster methods as a measurement of similarity from samples of ABA-treated vs. mock-treated *Arabidopsis* WT Col-0, *abi1td*, *mkkk17*, and *mkkk18* mutants. The data in the heatmap are the value of log2 (fold change). Red and blue indicate upregulation and downregulation of miRNAs, respectively.

**Figure 5:**
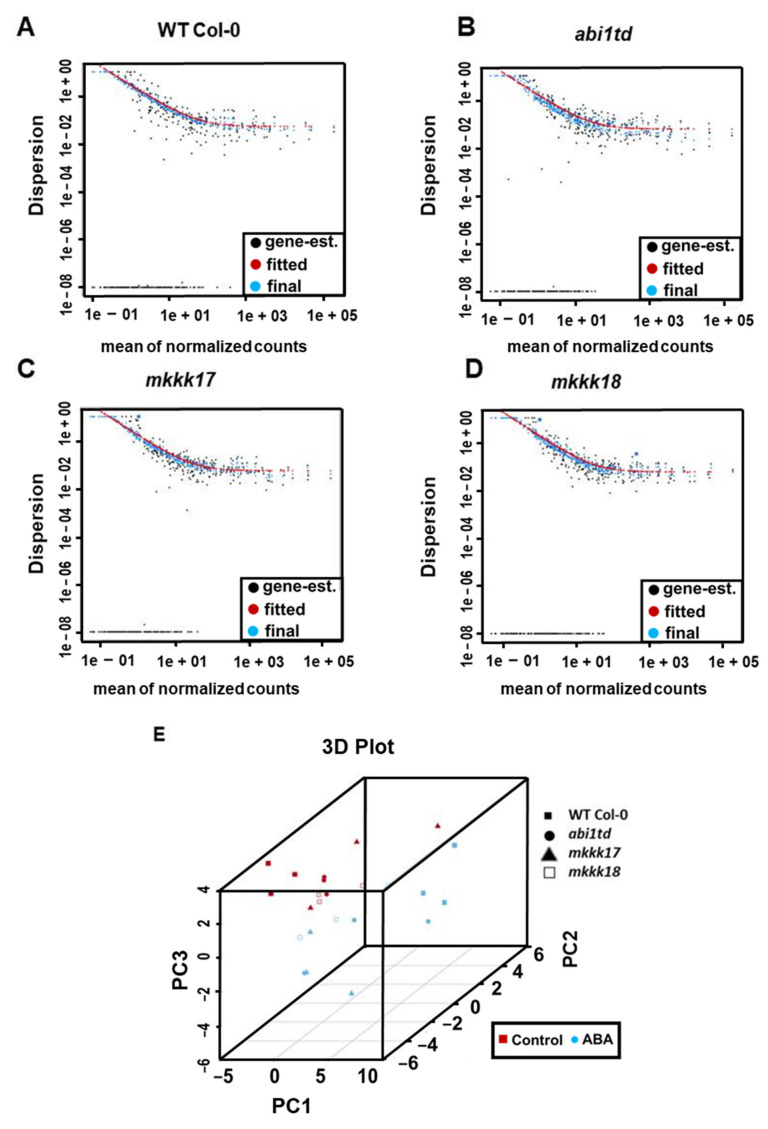
ABA-responsive small RNA clusters. Dispersion plot shows how the dispersion (variance) estimates are shrunk from the gene-wise values (black dots) toward the fitted estimates in (**A**) WT Col-0, (**B**) *abi1td*, (**C**) *mkkk17*, and (**D**) *mkkk18* mutants after ABA induction. Values in blue are the final values used in testing. (**E**) A three-dimensional PCA plot of small RNA-Seq libraries with or without ABA induction of all genotypes of *Arabidopsis* WT Col-0, *abi1td*, *mkkk17*, and *mkkk18* mutants.

In addition, we identified a few mature miRNAs in WT Col-0 as novel and named them ath-miRn-1, ath-miRn-2, ath-miRn-3, and so on. A few of the conserved miRNA families identified in our sequencing data were present in all libraries, as shown in Figure 4, where the heatmap demonstrates the miRNAs’ expression patterns in all genotypes. A heatmap was also prepared that shows sample-to-sample distances; samples with high similarity are preferentially clustered (Supplementary Figure S1). As shown in Figure 5A–D, a dispersion estimate plot was performed to illustrate the ‘fit’ of each miRNA’s dispersion to a curve that was constructed based on the assumption that those miRNAs of similar average expression level have a similar dispersion. Principal component analysis was performed, and the first three principal components were plotted to visualize the overall effect of experimental covariates and batch effects (Figure 5E).

### 2.3. Differentially Expressed miRNA Screening

After normalization of the reads as ‘reads per million’ and analysis of differentially expressed (DE) miRNAs (using the criterion of adjusted *p*-value < 0.05), 23 of the 85 known miRNAs were upregulated, whereas 45 were downregulated in WT. Among them, we identified ten novel miRNAs: six novel miRNAs were upregulated, and three were downregulated after ABA treatment (Supplementary Table S3). In mutant seedlings of the *abi1td* genotype, there were three DE known miRNAs (miR824-3p, ath-miR2111b-3p, ath-miR408-5p) upregulated after ABA treatment. In *mkkk17*, five of seven DE known miRNAs were upregulated, two were downregulated, and one novel miRNA (miRn-8) was highly downregulated in this mutant in response to ABA. In the *mkkk18* mutant, seven of nine DE known miRNAs were upregulated, one was downregulated, and one miRNA’s expression was considered unchanged after ABA treatment.

These results reveal that the expression levels of these miRNAs differ with genotype in ABA-induced samples, indicating that these DE miRNAs might be involved in the regulation of phase change in the presence of ABA due to its effect on post-transcriptional gene silencing.

**Figure 6:**
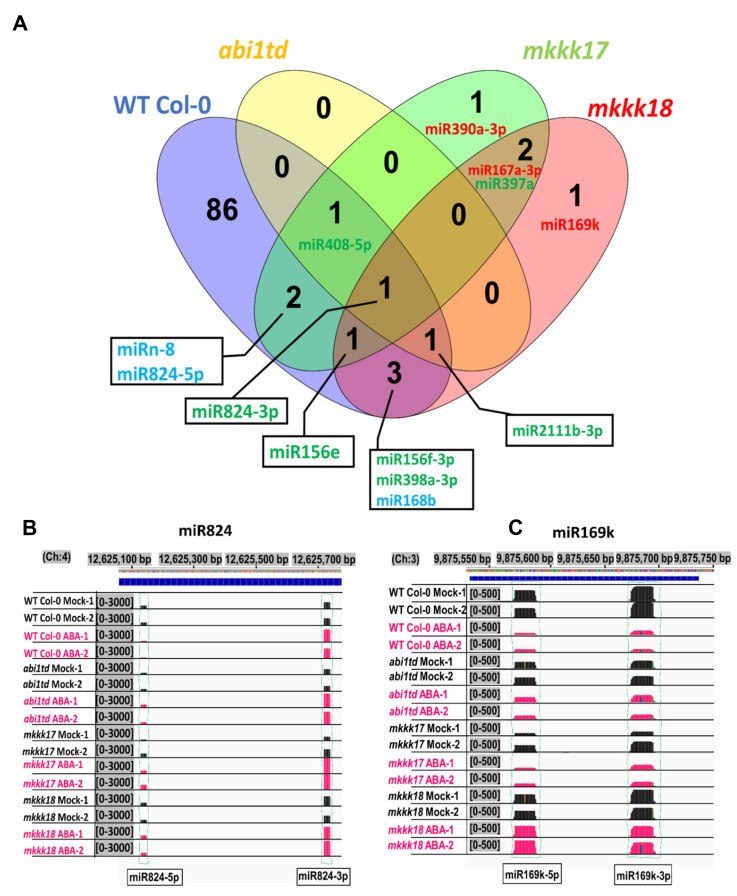
Common and specific miRNAs display differential expression between analyzed samples. Expression profiles and prediction comparison for miRNAs. (**A**) Venn diagrams show the common and specific differentially expressed known and novel up- and downregulated miRNAs or unchanged miRNAs expressed in *Arabidopsis* WT Col-0, *abi1td*, *mkkk17*, and *mkkk18* mutants before or after four hours of 100 µM ABA treatment (mock- and ABA-treated). The fold change (ABA-treated vs. mock) is log2N; log2N ≥ 1 is upregulated; between 0 < |log2N| < 1 is unchanged expression; and log2N ≤ −1 is downregulated. Adjusted *p*-values ≤ 0.05. Green-colored miRNAs show upregulated miRNAs in all genotypes. Red color shows downregulated miRNAs in all genotypes. Light blue color shows expression of miRNAs expressing differently in all genotypes. (**B**) miR824 and (**C**) miR169k show the abundance expressed in *Arabidopsis* WT Col-0, *abi1td*, *mkkk17*, and *mkkk18* mutants. Black bars indicate the number of aligned reads in mock samples, and red bars indicate the number of aligned reads in 4 h of ABA-treated samples; therefore, coverage at the specific positions and abundance can be compared between genotypes after 4 h of 100 µM ABA induction, and their genotype specificity can be observed after ABA in all genotypes with 3′, 5′-mature variants of these miRNAs. (Ch with numbers means chromosome number).

Among the statistically significantly regulated miRNAs, we identified representatives with genotype-specific expression patterns after ABA treatment. Some miRNAs were identified in both WT and one or more, but not all, mutant lines. Of the known and novel miRNAs that were differentially expressed in all genotypes, 86 miRNAs were WT-specific after ABA treatment (Figure 6A). One miRNA (miR408) was upregulated and was commonly expressed among WT, *abi1td*, and *mkkk17* genotypes. miRn-8 and miR-824-5p were differentially expressed in WT and *mkkk17* genotypes. DE miR156e was upregulated in WT, *mkkk17*, and *mkkk18* genotypes. miR2111b-3p was differentially expressed in WT, *abi1td*, and *mkkk18*. Two miRNAs (miR167a-3p, miR397a) were expressed commonly only in *mkkk17* and *mkkk18* after ABA. In other cases, miRNAs were identified in mutants only; for instance, DE miR390a-3p and miR169k were highly downregulated only in *mkkk17* and *mkkk18* mutants, respectively, as shown in Figure 6A–C.

**Figure 7:**
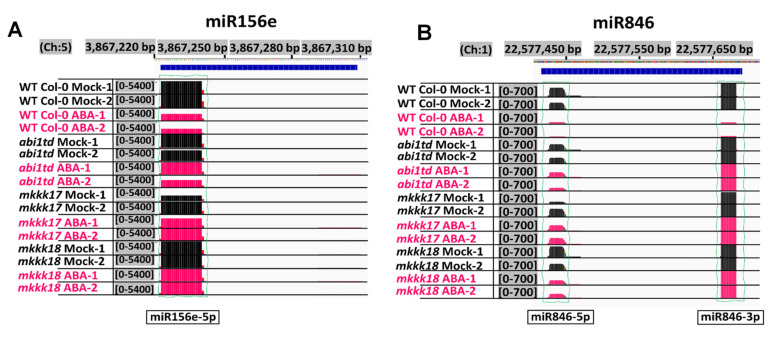
Expression profiles of the known miRNAs’ abundance in *Arabidopsis* WT Col-0, *abi1td*, *mkkk17*, and *mkkk18* mutants with or without ABA induction. These show the abundance of miRNAs (**A**) miR156e and (**B**) miR846 expressed in *Arabidopsis* WT Col-0, *abi1td*, *mkkk17*, and *mkkk18* mutants. Black bars indicate the number of aligned reads in mock samples and red bars indicate the number of aligned reads in 4 h of 100 µM ABA-treated samples; therefore, coverage at the specific positions and abundance can be compared between genotypes after 4 h of ABA induction. Strong or weak expression can be observed for these miRNAs in all genotypes for the 3′, 5′-mature variants of these miRNAs. (Ch with numbers means chromosome number).

To further understand the post-transcriptional changes occurring after ABA treatment in WT and mutants, further analyses of the differentially expressed miRNAs were carried out. For example, the number of reads in ABA-treated libraries showed that the abundance of miRNA846 and miR156e in WT was lower than in the three mutants (Figure 7A,B). These results might reflect the significant impact of ABA on the post-transcriptional regulation of miRNA target genes and that these miRNAs have common regulatory roles in the core ABA signaling pathway.

### 2.4. Expression Profiling by qRT-PCR of Known and Novel miRNAs

**Figure 8:**
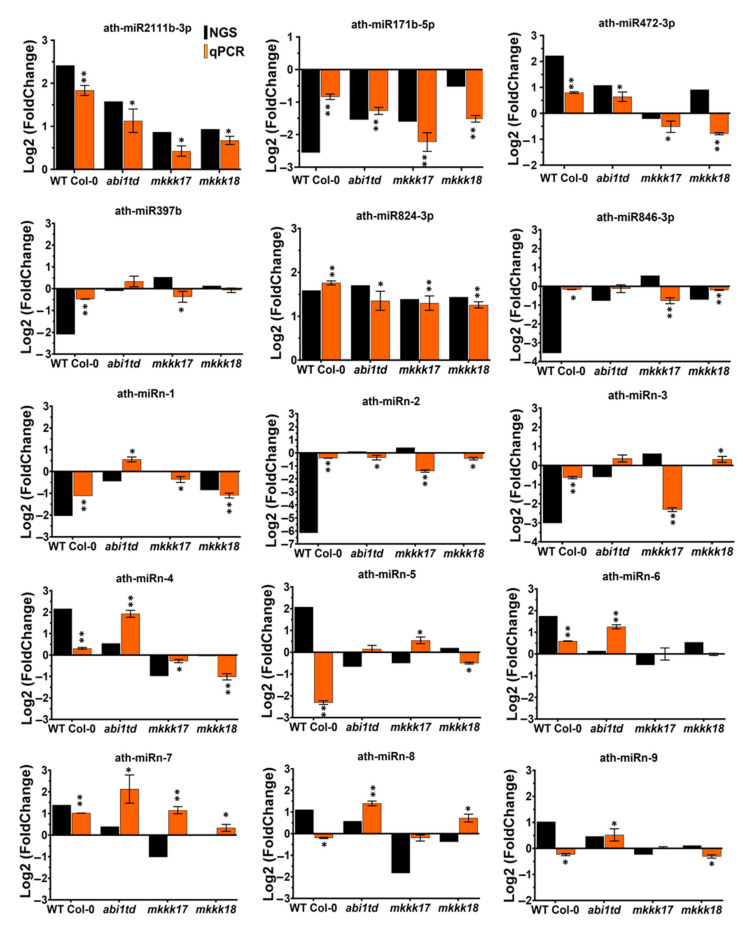
Validation and comparison of RNA sequencing results by real-time PCR. Comparisons between real-time validation and gene expression profiling data of randomly selected known and novel miRNAs in *Arabidopsis* WT Col-0, *abi1td*, *mkkk17*, and *mkkk18* mutants before or after four hours of 100 µM ABA treatment (mock- and ABA-treated). The *x*-axis represents genotype names, while the *y*-axis represents the relative expression level of miRNAs. The expression levels of miRNAs are normalized to the level of *SnoR66*. Black bars represent the expression from log2 (fold change) of the NGS data, while orange bars represent the qPCR log2 (fold change) values. Data from qRT-PCR are means of three biological replicates with each having three technical replicates. Error bars represent ±SD (standard deviation) from triplicates. Student’s *t*-test was performed on mock-treated and four hours of 100 µM ABA-treated results of qRT-PCR, and the statistically significant treatments are marked with * (*p*-value ≤ 0.05), ** (*p*-value ≤ 0.001).

To validate the sequencing data, 15 miRNAs (six known miRNAs and nine novel miRNAs) were selected at random and subjected to qRT-PCR using TaqMan^®^ miRNA assays (ABI, Life Technologies, Carlsbad, CA, USA). All 15 miRNAs were tested against RNA samples from mock- and ABA-induced WT Col-0, *abi1td*, *mkkk17*, and *mkkk18* mutants. Of the six known miRNAs, after ABA treatment, three were upregulated and three downregulated across all genotypes, while three of the novel miRNAs were downregulated and the remaining six upregulated. In almost all cases, the expression levels were consistent with the sequencing data: although the log_2_ fold changes for the various genotypes were occasionally different to those suggested by the NGS data, the trends were generally consistent, although not in all cases (Figure 8). Thus, qRT-PCR data for ath-miR2111b-3p, ath-miR824-3p, ath-miR171-5p, ath-miR472-3p, ath-miRn-4, ath-miRn-6, and ath-miRn-8 broadly matched the NGS data in almost all genotypes. However, some miRNAs showed differences between the qRT-PCR and NGS data—for example, ath-miR846-3p, ath-miRn-1, ath-miRn-2, ath-miRn-3, ath-miRn-5, ath-miRn-7, and ath-miRn-9, which were upregulated after ABA treatment according to the sequencing data but were downregulated according to the qPCR data, and vice versa (Figure 8). This shifting trendpossibly occurred because we used different or newly prepared biological replicate samples instead of using the same samples for NGS.

### 2.5. Target mRNA Prediction, 5′ RLM-RACE Validation, and Target Gene Expression by qRT-PCR

**Figure 9:**
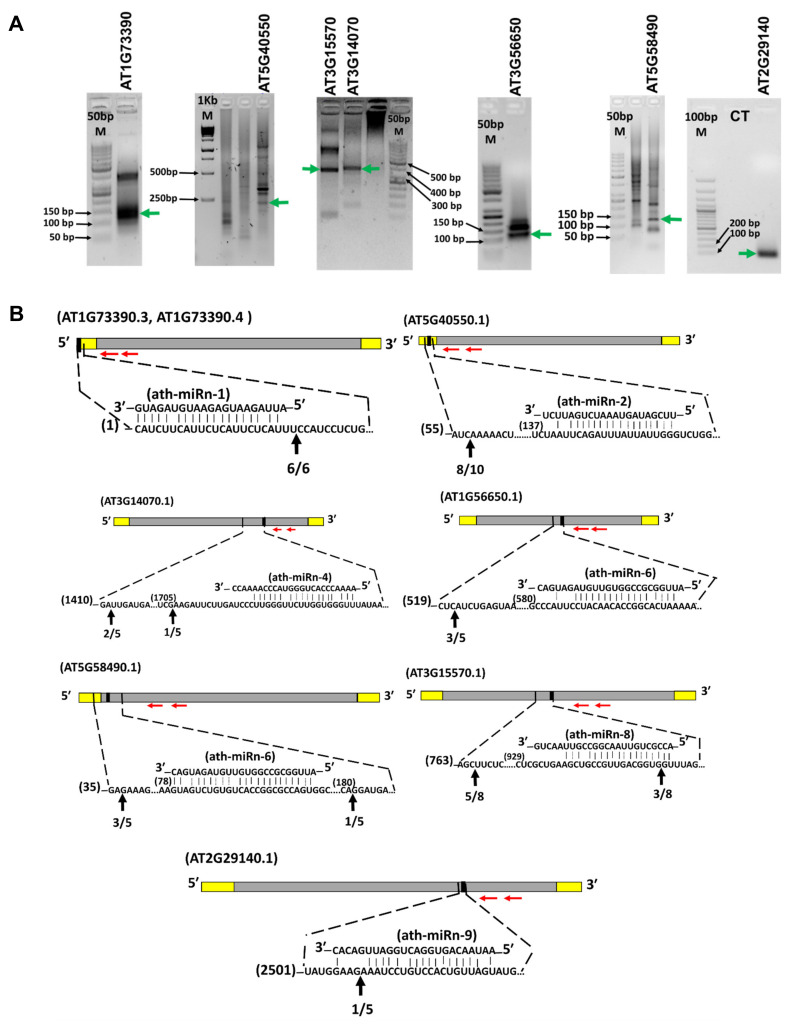
miRNA mapping and cleavage site determination through 5′ RLM RACE. (**A**) Gel images of 5′ RLM-RACE showing the amplified products (RLM-RACE second round PCR products) after consecutive PCR reactions. The migrating band in each lane (green horizontal arrows) was purified for DNA sequencing. CT: negative control for PCR; M: DNA molecular weight size marker. (**B**) The targeted mRNA section and miRNA sequences, along with mismatch(es), if any, are shown as the empty region or with dotted line. Grey boxes show CDS regions of miRNA target genes. Yellow boxes show 5′, 3′ UTR ends of miRNA target genes. Black boxes show the predicted region of miRNA target site. The enlarged portion shows the pairing between miRNAs and the target sites. Each top strand depicts an miRNA, and each bottom strand depicts complementary target site on the anti-parallel miRNA. The 5′ ends of the cleaved product determined by sequencing are indicated by the vertical arrowheads, along with the numbers of clones analyzed for each target gene. The horizontal red arrowheads indicate the gene-specific primer sites used for 5′ RLM-RACE. Numbers in brackets show nucleotides position of target gene cDNAs.

Identification of mRNA targets is crucial for the understanding of miRNA function. Direct target genes for these novel miRNAs were predicted using psRNATarget (Table 3). Of these predicted target genesWe used 5′ RLM-RACE to validate the miRNA cleavage sites in target mRNAs. All 5′ RLM-RACE PCR products were analyzed on agarose gels Figure 9A). purified, cloned, and sequenced. We identified cleavage sites for six of the novel miRNAs in seven target genes, which supports the view that these genes are the direct targets of the corresponding miRNAs in *Arabidopsis*. Two other cleavage sites were located outside the complementary region. These findings verified cleavage sites in target genes AT1G73390.3/AT1G73390.4 for ath-miRn-1, AT5G40550.1 for ath-miRn-2, AT3G14070 for ath-miRn-4, AT1G56650, AT5G58490 for ath-miRn-6, AT3G15570 for ath-miRn-8, and AT2G29140 for ath-miRn-9. Among these target genes, a cleavage site for ath-miRn-9 was detected in the predicted miRNA complementary region, while for ath-miRn-8, cleavage sites were detected in the predicted miRNA complementary region and also upstream of this region (Figure 9B).

For the other four target genes, cleavage sites were detected up- or downstream of the predicted complementary region, perhaps due to a secondary siRNA in the 21 nt register with a cleavage site for miRNAs, as reported previously by other researchers [43,44]. These novel miRNAs are likely to affect post-transcriptional modifications of their target genes by mRNA cleavage in *Arabidopsis* after ABA treatment.

**Figure 10:**
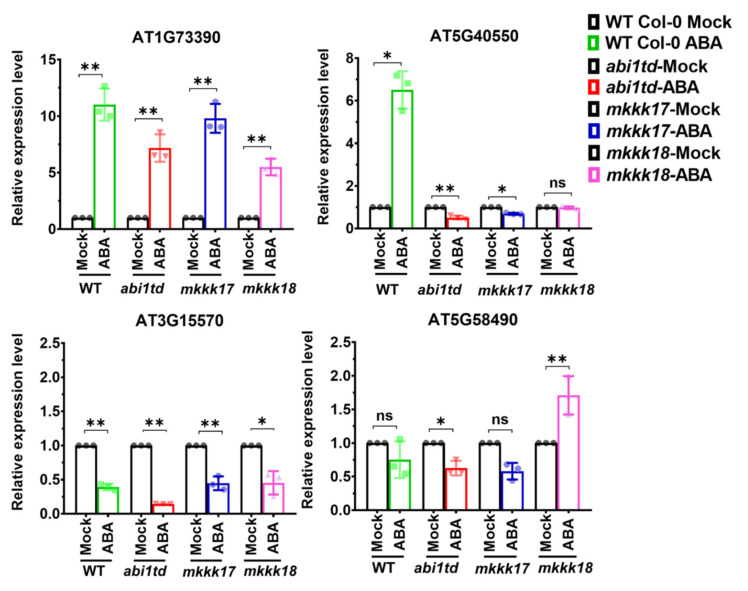
Relative transcript abundance of selected target genes of novel miRNAs identified as ABA-responsive in this study by RT-qPCR. Target gene expression profiling data of randomly selected target genes of novel miRNAs in *Arabidopsis* WT Col-0, *abi1td*, *mkkk17*, and *mkkk18* mutants before or after 4 h of 100 µM ABA treatment (mock- and ABA-treated). The *x*-axis represents genotype names, while the *y*-axis represents the relative expression level of miRNA target genes. The expression levels are normalized to the level of 18S as internal control. Data from qRT-PCR are means of three biological replicates, each of which has three technical replicates (*n* = 9). Error bars represent ±SD (standard deviation) from triplicates. Student’s *t*-test was performed on mock- and ABA-treated results of qRT-PCR, and statistically significant treatments are marked * (*p*-value ≤ 0.05), ** (*p*-value ≤ 0.001), or ns (non-significant).

Functional annotation of the predicted target genes of differentially expressed novel miRNAs is shown in Table 3. To test whether novel miRNA target genes are regulated by ABA, we performed qPCR: several showed positive ABA-responsive expression in *Arabidopsis* WT Col-0, *abi1td*, *mkkk17*, and *mkkk18* mutants before or after 4 h of 100 µM ABA treatment (mock- and ABA-treated) (see Figure 10). The results confirmed that these selected miRNA target genes are significantly regulated by ABA in almost all genotypes.

### 2.6. Functional Enrichment of miRNA Target Genes

Putative targets were predicted for the miRNAs that were differentially expressed using the online server psRNATarget website. After filtering, unique putative target genes of upregulated and downregulated miRNAs were obtained across all genotypes (Supplementary Tables S9–S12). Gene ontology (GO) classification was used to annotate these target genes in the cellular process, metabolic process, and biological mechanism categories. Analysis showed that upregulated miRNA target genes were more enriched in the biological processes of ‘phosphorylation’ and ‘protein phosphorylation’ in WT Col-0 compared to *abi1td* and *mkkk17*, and the target genes in these categories were not present at all in the *mkkk18* mutant after ABA treatment. While the upregulated miRNA target genes in WT were more enriched in the biological process of ‘transcription, DNA-templated’ than in the *mkkk18* mutants, no enrichment was observed for the upregulated miRNA target genes in the *abi1td* and *mkkk17* mutants after ABA treatment. Interestingly, the top 10 enriched roles of the target genes of up- and downregulated miRNAs, i.e., the molecular processes ‘ion binding’, ‘protein binding’, ‘ATP binding’, ‘nucleotide binding’, ‘metal ion binding’, ‘DNA binding, ‘transferase activity’, ‘nucleic acid binding transcription factor activity’, and ‘kinase activity’ were much more enriched in WT Col-0 than in the *abi1td*, *mkkk17*, and *mkkk18* mutants after ABA treatment (Supplementary Tables S13 and S14). All biological and molecular processes, including signaling, response to stimulus, developmental process, and reproductive process, were found to be regulated by the target genes of DE miRNAs after ABA treatment in all genotypes, which suggests that miRNAs in *Arabidopsis* have a broad regulatory role in ABA signaling.

The target genes of DE miRNAs were mapped in Expath 2.0 to Kyoto Encyclopedia of Genes and Genomes (KEGG) pathways. Previously, ABA has been reported to regulate lysine catabolism in *Arabidopsis* [45], and our KEGG analysis of the predicted target genes of upregulated miRNAs showed that the ‘Lysine degradation’ pathway was significantly enriched, but only in WT and the *mkkk18* mutant. Depletion of glutathione enhances ABA- and methyl jasmonate-induced stomatal closure in *A. thaliana* [46] and glutathione peroxidase functions as both a redox transducer and a scavenger in the ABA and drought stress responses [47]. Furthermore, ABA participates in the antagonistic modulation of ethylene and in the accumulation of reactive oxygen species [48]. In the KEGG analysis of the predicted target genes of upregulated miRNAs, ‘Glutathione metabolism’ and ‘Ascorbate and aldarate metabolism’ were significantly enriched in WT, *abi1td*, and *mkkk18* mutants after ABA treatment. It has been proposed that mechanisms independent of ABA or proline feedback play a predominant role in the transcriptional regulation of proline metabolism during low water potential and stress recovery [49]. The predicted target genes of upregulated miRNAs were enriched after ABA treatment in the ‘Arginine and proline metabolism’ KEGG pathway in all genotypes, while the predicted target genes of downregulated miRNAs were not observed in all genotypes. The involvement of ABA in thiamine biosynthesis under stress conditions in *Arabidopsis* has been confirmed [50]. KEGG analysis showed that target genes of upregulated miRNAs were enriched in the ‘thiamine metabolism’ pathway, but only in WT. Moreover, after ABA treatment, the ‘alanine, aspartate, and glutamate metabolism’ KEGG pathway was found in upregulated miRNAs predicted target genes in the *mkkk18* mutant (Supplementary Tables S15 and S16). Overall, the results suggest that, via its effect on various miRNAs, ABA regulates the expression of many target genes in *Arabidopsis* that specifically modulate a number of important biochemical pathways.

## 3. Discussion

In *Arabidopsis* and other plants, miRNAs are involved in a wide range of physiological processes, such as phytohormone signaling, biotic and abiotic stress responses, plant development, and nutrient signaling pathways. Specific examples include the brassinosteroid response and the response to blue light in *Arabidopsis*, and pollen development and the heat stress response in *Solanum lycopersicum*. Other types of non-coding RNA include sesame, as well as in the cold response in grapevine, the drought response in mulberry, and the drought-responsive multi-tiered regulatory network in drought-tolerant rice [23,30,51,52,53,54,55,56]. However, a role for miRNAs in regulating the MAPK cascade that operates downstream of the core ABA signaling pathway has not been confirmed in *Arabidopsis*. Here, we identified a number of miRNAs that are likely involved in this cascade and thus will be the subject of further detailed research.

An average of more than 8–10 million clean reads for each sample of sRNA was obtained by high-throughput sequencing, and known and novel miRNAs from the control and ABA-treated libraries were identified by bioinformatics. Small sRNA reads of 24 nt were the most predominant sequence length, followed by 21 nt sRNAs, which is consistent with the results of high-throughput sequencing in other plants, such as *Dimocarpus longan* Lour [26] and tea plant [57]. Our results are in accordance with earlier reports from different plants, which show that 24 nt sRNAs are a highly abundant category of sRNA reads [58,59,60]. miRNA length is important for alignment with the RNA-induced silencing complex (*RISC*), which results in the degradation of the target mRNA or inhibition of its translation.

The predicted 276, 388, 262, and 167 known and 10 novel miRNAs were identified in the respective WT Col-0, *abi1td*, *mkkk17*, and *mkkk18* genotypes (both control and ABA-induced samples). Importantly, the highest number of miRNAs was identified in the *abi1td* mutant (see Figure 2), suggesting that increased phosphorylation status significantly affects miRNA expression and abundance. All identified miRNAs were clustered into different miRNA families. The miR166 family members were the most abundant group across all genotypes, suggesting a significant role for these miRNAs in the growth and development of *Arabidopsis* during ABA treatment. These findings suggested that the identified miRNAs regulate various functions in *Arabidopsis* during the presence of ABA induction.

In our study, DE novel miRNAs identified in WT were validated by qPCR, and cleavage sites of novel miRNAs in their target genes were also confirmed. However, 15 randomly selected (6 known and 9 novel) miRNAs were confirmed by stem-loop RT-PCR (Figure 8). Nevertheless, we were able to determine a number of properties of these ABA-regulated miRNAs in *Arabidopsis*, particularly in the identification of their putative target genes, which is facilitated in plants by the perfect or near-perfect complementarity between miRNAs and their target mRNAs [61]. Indeed, for six miRNAs, we were able to validate the corresponding targets by determining mRNA cleavage sites by 5′ RACE (Figure 9). Interestingly, expression of all six of these miRNAs was regulated by ABA (Figure 8) and their targets play important roles. For example, the protein encoded by AT1G73390 is one of an ABA-regulated set of genes that interacts with the *SnRK1* complex involved in energy homeostasis [62]. The transcription factor (TF) gene *MYB75* (AT1G56650) regulates various biosynthetic pathways and is involved in the responses to light [63] and to fungus [64], for instance, and we confirmed its mRNA as a target of a novel miRNA by 5′ RACE (Figure 9); this means that *MYB75* could be regulated by ABA via the novel miRNA (ath-miRn-6) in *Arabidopsis*. Another novel miRNA target gene highlighted in our study is *PUM3* (AT2G29140), whose cognate protein was previously reported to be a mRNA-binding protein [65,66]. Thus, we propose that these novel miRNAs could be involved in many functions controlled by various mechanisms in *Arabidopsis*.

miRNA-mediated stress responses are often closely associated with target TF genes that transcriptionally [67] regulate downstream stress-responsive or stress-defensive genes. Several miRNA target mRNAs are known to encode TFs and, among these, many are implicated in hormonal signaling. Such relationships between miRNAs and hormonal signaling components may be crucial for plant development. For example, in cotton, MAPK families are of great importance in coping with drought and salinity stress, and many conserved and novel miRNAs have been identified that target specific gene members [68]. The miRNA miR846 is regulated by ABA treatment, and both miR846 and ABA regulate a gene for a mannose-binding lectin superfamily protein [7]. We found that miR846 is more downregulated in WT seedlings and less downregulated in mutant line *mkkk18* than in WT (Figure 7B). This means that miR846 is more abundant after ABA treatment in the *mkkk18* mutant and suggests that mannose-binding lectin superfamily protein accumulation must be lower in *mkkk18* than in WT. It has been reported that miR172b controls the transition to autotrophic development that is inhibited by ABA in *Arabidopsis*, and activation of miR172b and *SNZ* is completely abolished by ABA and osmotic stress [69]. Salt and ABA treatment increase the expression of miR399f, and *Arabidopsis* plants overexpressing miR399f show enhanced tolerance to salt stress and exogenous ABA, while mRNA levels of *ABF3* and *CSP41b* decrease markedly in miR399f-overexpressing plants under salt stress and in response to ABA [70]. Our data show that ath-miR172b is downregulated and ath-miR399f upregulated in WT Col-0, which supports previous studies. Thus, these miRNAs likely exert crucial functions in the morphological and metabolic adaptation of plants to ABA treatment.

Recent studies have also revealed that miRNAs regulate adaptive responses to nutrient deficiency in plants and drought stress tolerance [71,72]. For example, miR169, which is encoded by multiple loci, regulates At1g54160 (*NFYA5*) expression and tolerance to drought stress [73]. Interestingly, in our experiments, we observed the genotype-specific expression of eight miR169 precursors (Supplementary Tables S1–S4), which were significantly downregulated, with the exception of at-miR169k, which was upregulated in *mkkk18* after ABA treatment (Figure 6C). We found that ath-miR169b-3p, b-5p, f-3p, f-5p, g, h, k, and n were expressed and downregulated in WT Col-0. Ath-miR169f-3p, g, k, and n were expressed in *mkkk17*, while ath-miR169b-5p, f-3p, and g were identified and downregulated in the *abi1td* mutant. Our results suggest, therefore, that degradation of miR169 gene targets depends on the conditions and could be achieved by different members of the miR169 family.

The signaling and translocation of miRNAs during nutrient starvation responses and specific developmental events have already been reported in various plant species, including *Arabidopsis* [59,71,74,75]. The ath-miR171-*SCL* module is critical for mediating GA-DELLA signaling, and *SCL27* has an important role in inhibiting chloroplast development before cell expansion [76]. In our data analysis, it was very interesting to note that the expression patterns of some miRNAs were genotype-specific after ABA treatment in mutants compared to WT seedlings; for example, ath-miR171b-5p was more downregulated in *abi1td* and *mkkk17* mutants than in WT. This suggests to us that decreased expression of this miRNA might affect GA-DELLA signaling in the presence of ABA.

Drought and salinity cause nutritional imbalance in plants [77]. At3g27150, which encodes a galactose oxidase/kelch repeat superfamily protein and is induced in phosphate-starvation conditions, is a regulatory target of ath-miR2111b [78]. *Ascophyllum nodosum* extract and NaCl modify the expression of ath-miR2111b and its target At3g27150, suggesting a role for *A. nodosum* extract in phosphate homeostasis [79]. We found the expression of ath-miR2111b to be modest, but statistically significant; it was upregulated in WT and the *abi1td* mutant, but almost unchanged in *mkkk17* and *mkkk18*. This suggests that the expression of miR2111b target genes must be relatively higher in these two genotypes than in WT, which could have functional significance for ABA signaling. Patterns of miR2111b expression identified by qRT-PCR were largely in accordance with the sRNA sequencing results, validating our RNA-Seq data. It has been reported that miR472-3p is induced by copper in *Arabidopsis* [80]; miR472-3p is upregulated in WT, but its expression is lower in the mutants, resulting in increased expression of its target genes, especially in *mkkk17* and *mkkk18*. Distinct miRNAs exert multiple functions in plant stress responses. Thus, as well as involvement in the Pi-signaling response, these miRNAs might also mediate ABA responses, conferring improved salt and drought tolerance on plants by post-transcriptional regulation of target genes.

There are various miRNAs that are reported to be involved in the physiological functions of plants as well as various plant stresses: for example, according to TAIR annotation, AT2G02850 encodes plantacyanin (a copper protein) and is involved in anther development and pollination. AT2G02850 was experimentally confirmed to be cleaved by miR408 in *Arabidopsis* [81], and its expression correlates with the abundant levels of miR408 in flowers [82]. Our data show that ath-miR408-5p is more highly induced in *abi1td* and *mkkk17* compared to WT Col-0 and that ath-miR408-3p is upregulated in *mkkk18* compared to other genotypes in response to ABA, indicating that these miRNAs might be more critical for mediating the effect of ABA in *mkkk18* than in WT. In miR156e-3p-overexpressing transgenic plants, the expression of its target gene *SPL1* is decreased in *Arabidopsis* [83], and *AP2* directly promotes the expression of miR156e, which blocks the floral transition by repressing SPL genes [84]; our sequencing data show that miR156e is induced to some extent in all genotypes except the *abi1td* mutant after ABA treatment (Figure 7A). ath-miR397a and ath-miR397b are expressed in flowers and seedlings [82], and 24-epibrassinolide (EBR) upregulates ath-miR397a [85]. Our analysis shows that ath-miR397a is significantly upregulated in mutants of *mkkk17* and *mkkk18*, whereas ath-miR397b is highly downregulated by ABA in WT only; qRT-PCR confirmed these expression patterns (Figure 8), which demonstrates that these miRNAs could be mediating ABA signal transduction during these developmental stages. Previous experimental studies have shown that overexpression of only ath-miR167a of the four ath-miR167s arrested flower development, similarly to mutation of the target arf6-2 and arf8-3 genes [86]; the authors therefore propose that there is a prime requirement for miR167a to be conserved in diverse plant species. Interestingly, in our analysis of mature miRNAs, ath-miR167a-3p expression was reduced only in the mutants *mkkk17* and *mkkk18* and not in WT, while ath-miR167b was induced by ABA only in WT Col-0 seedlings. This shows that miR167-mediated post-transcriptional regulation is an important regulatory pathway involved in the regulation of flower development and the ABA response.

miR168 targets the mRNA of *AGO1* [10], and NaCl leads to the upregulation of ath-miR168 [87], as does six hours of NaCl treatment [79]. It is also evident from our data that DE ath-miR168a-3p is highly induced in WT Col-0 only, while DE ath-miR168b is upregulated only in the *mkkk18* mutant after ABA treatment. The miRNA390-dependent transacting small interfering RNA pathway is regulated by auxin [19], and the targets of this miRNA have been validated [88]; miRNA390 is also hypoxia-responsive [58]. In response to ABA treatment, miR390a was downregulated in *mkkk17*, but was unchanged in WT and other mutant lines, which suggests that the target gene of this miRNA might be overrepresented in *mkkk17*. Taken together, the data for these DE miRNAs indicate possible crosstalk between the auxin and ABA signaling pathways by regulation of the respective miRNA target genes.

**Figure 11:**
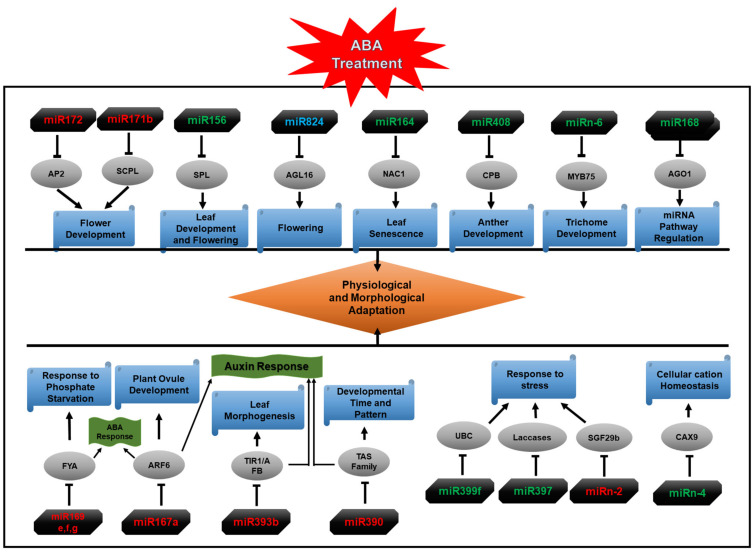
A hypothetical model of regulatory networks of ABA-responsive miRNAs and their target genes in *Arabidopsis*. Possible roles of known and novel ABA-induced miRNAs in different biological processes in *Arabidopsis* based on Gene Ontology (GO) analysis. Downregulated ABA-responsive miRNAs are shown in red, while those shown in green represent upregulated ABA-responsive miRNAs identified in this study. miRNAs labeled in green are upregulated in all genotypes; those in red are downregulated in all genotypes; miRNAs labeled in light blue are expressed differently in all genotypes. Grey ovals show target genes of miRNAs. Block/inhibitory arrows show the regulation of target gene by miRNA.

Plant growth and development are regulated by complex gene networks. Target gene identification and, subsequently, GO analysis and KEGG pathway assignment linked our panel of miRNAs and their target genes with various biological processes, cellular components, and molecular functions (Supplementary Tables S15 and S16). On the basis of this analysis, a hypothetical model of regulatory networks of ABA-responsive miRNAs and their target genes in *Arabidopsis* can be proposed (Figure 11). These findings suggest that the miRNAs identified in our study regulate a range of functions in response to ABA.

## 4. Materials and Methods

### 4.1. Plant Materials and Sample Preparation for Sequencing

Seeds of WT *Arabidopsis thaliana*, Columbia ecotype (Col-0), as well as *abi1td*, *mapkkk17td*, and *mapkkk18td* mutants were used in this study. The *abi1td* [89], *mkkk17* (SALK_080309C), and *mkkk18* mutant lines were created by T-DNA insertion as described previously [4]. After two days of stratification at 4°C, the plants were grown in Half Murashige and Skoog Basal Medium supplemented with sucrose. Seedlings were grown as described [89] for two weeks, after which they were treated with 100 μM ABA (dissolved in methanol) or an equivalent mock treatment, and the petri plates were then incubated for 4 h in a growth chamber. The harvested plant materials were frozen in liquid nitrogen and stored in a −80 °C freezer until further use. For each condition (mock- and ABA-treated), three independent biological replicates were used, and small RNA was extracted using TRIzol^®^ Reagent (Invitrogen, Carlsbad, CA, USA) according to the manufacturer’s protocol (Direct-zol RNA Miniprep; ZYMO Research Corporation, Irvine, CA, USA). ABA application was confirmed by the amplification of ABA-related marker mRNAs. Induction or downregulation of marker mRNAs was tested by real-time PCR analysis. All measurements were carried out in three biological replicates. An Agilent 2100 Bioanalyzer System (Agilent, Santa Clara, CA, USA) was used to confirm the RNA integrity (>8) of the miRNA samples.

### 4.2. Sequencing and Processing of Reads

Macrogen Ltd., Seoul, South Korea, carried out miRNA sequencing using the TruSeq Small RNA Library Prep Guide (Part #15004197 Rev. G protocol) on the Illumina HiSeq2500 platform to generate 50 bp paired-end reads. Adapters were clipped, and reads with no adapters and reads less than 16 nt long were discarded. The reads were then filtered for quality using the FASTX-toolkit, requiring that at least 95% of nucleotides had a Phred quality scores of 20 or more. Read cataloging was performed using a mirPRo stand-alone pipeline [90]. Identification of miRNAs was performed with ShortStack v-3.3 [91] with default parameters, except that the presence of miRNA* was not strictly required. As the miRNAs were identified for each sample independently, a unique set of miRNAs was obtained by merging their coordinates with the Bedtools [92] merge utility (https://bedtools.readthedocs.io/en/latest/content/tools/merge.html, accessed on 30 October 2018). Additionally, we kept only miRNAs identified in at least two distinct samples and with a pre-miRNA sequence not shorter than 40 nt. miRNA* sequences were filtered out using Bedtools to find unique miRNA loci. The novel precursor sequences were used to construct secondary structures using RNA structure version 6.2 software (https://rna.urmc.rochester.edu/RNAstructure.html) with the default settings. Mature miRNAs were annotated further using the miRbase database (version 22 http://www.mirbase.org/, accessed on 30 October 2018) to identify both conserved and novel miRNAs. The raw sequencing data (20 GB) were deposited at NCBI GEO with the accession number GSE172377.

### 4.3. Differential Expression Analysis

The raw counts were analyzed using the DESeq2 package [93] to find differentially expressed mature miRNAs. In our analysis, a false discovery rate of 0.05 was implemented, and any mature miRNA, which was lower than the *p*-adjusted value, was defined as significantly down- or upregulated. To prepare the Venn diagram, an online webtool (http://bioinformatics.psb.ugent.be/webtools/Venn/) (accessed on 5 November 2018) was used to identify differentially expressed miRNAs that were shared between mock- and ABA-treated samples. Similarly, the effect of mutants was investigated by comparing untreated control samples of mutants (*abi1td*, *mkkk17*, *mkkk18*) with WT. The interaction effect between genotypes was studied by principal component analysis to find the variance among samples.

### 4.4. Validation of miRNA Expression with Stem-Loop qRT-PCR

Total RNA from three independent biological replicates was extracted using Tri-reagent according to the manufacturer’s protocol (Direct-zol RNA Miniprep kit; ZYMO Research Corporation, Irvine, CA, U.S.A). RNA samples were treated with DNaseI provided in the RNA Miniprep kit to remove genomic DNA. For qPCR analysis, 10 ng total RNA was used to synthesize cDNA from small RNAs. A total of 1.5 µL 10× reverse transcription buffer, 0.15 µL 100 mM deoxynucleotide triphosphates, 0.19 µL RNase inhibitor (20 units/µL), 1 µL TaqMan^®^ MicroRNA Reverse Transcriptase using TaqMan^®^ MicroRNA Reverse Transcription Kit (ABI, Life Technologies, Carlsbad, CA, USA), according to the manufacturer’s protocol, along with 3 µL stem-loop reverse transcription primer (ABI, Life Technologies, Carlsbad, CA, USA) in a 15 µL reaction mixture was used. The TaqMan^®^ miRNA assays were performed using TaqMan^®^ Universal PCR Master Mix II with uracil N-glycosylase (ABI, Life Technologies, Carlsbad, CA, USA) according to the manufacturer’s protocol. The reaction was performed at 50 °C for 2 min (for optimal UNG enzyme activity), at 95 °C for 10 min, followed by 40 cycles of 95 °C for 15 s, and 60 °C for 60 s. Ct values for all miRNA primary transcripts and other transcript cDNAs were normalized to the SnoR66 and Actin8 TaqMan Assay cDNA fragment Ct value; both had the same trend of expression of miRNAs, so the qPCR results show results using SnoR66 as endogenous control. Differences in the relative transcript levels were calculated using the 2^−ΔΔCt^ method. For each sample, the PCR amplification was repeated three times, and the average values of 2^−ΔΔCt^ were used to determine the differences in gene expression by Student’s *t*-test. Three biological replicates were performed with similar results, and one replicate is shown. TaqMan^®^ miRNA assay details are available in Supplementary Table S1.

### 4.5. Target Validation by 5′ RLM-RACE-PCR and miRNA Target Gene Expression by qRT-PCR

Target mRNA cleavage sites for several novel miRNAs were experimentally mapped by RNA ligase-mediated rapid amplification of cDNA ends (5′ RLM-RACE). A modified procedure for 5′ RLM-RACE was followed using the FirstChoice RLM-RACE Kit^®^ (ThermoFisher Scientific/Invitrogen, Carlsbad, CA, USA). To examine miRNA-directed cleavage of predicted targets in vivo, we isolated total RNA using Tri-reagent, as described above, from seedlings of A. thaliana. We employed the 5′ RLM-RACE kit to amplify the 5′ UTR of target gene mRNAs. These target genes were selected based on the prediction value (from 0 to 3.5) of novel miRNA target genes. Combinations of three different primers were used in the 5′ RLM-RACE experiments as listed in Supplementary Table S1. Total RNA (10 µg) was used without treatment with calf intestine phosphatase (CIP) and tobacco acid pyrophosphatase (TAP), which is not required for miRNA-cleaved mRNAs. RNA was then ligated with an RNA adaptor (45 nt long) using T4 RNA ligase. Using the above as a template for RT-PCR, we amplified the 5′ ends of mRNAs using an miRNA-target-specific primer and an adapter primer in first and second round PCR according to the manufacturer’s protocol. The final RLM-RACE products were analyzed on agarose gels and purified using a DNA gel extraction kit^®^ (ThermoFisher Scientific/Invitrogen, Carlsbad, CA, USA) according to the manufacturer’s instructions. The amplicons were then cloned into the TA cloning vector, pGEMT-easy (Promega, Madison, WI, USA), and sequenced.

To quantify the identified target genes of novel miRNAs, qRT-PCR was performed using a published protocol [89] with minor modifications. Relative quantification of gene expression was calculated using 18S as an internal control. Differences in the relative transcript levels were calculated using the 2^−ΔΔCt^ method. For each sample, the PCR amplification was repeated three times, and the average values of 2^−ΔΔCt^ were used to determine the differences in gene expression by Student’s *t*-test. Three biological replicates were performed for all target genes. All primers used in the qRT-PCR analyses are available in Supplementary Table S1.

### 4.6. Target Gene Identification, Gene Ontology, and KEGG Pathway Analysis

The putative target genes of upregulated (|log_2_(fold change)| > 1 or downregulated |log_2_ (fold change)| < −1) known and novel miRNAs were predicted using the online server psRNATarget (http://plantgrn.noble.org/psRNATarget/) [94] with default parameters. GO and KEGG enrichment analyses of the predicted target genes for all genotypes were performed separately for molecular function, biological process, and cellular component. GO enrichment and KEGG pathway analyses were performed using the ExPATH database (http://expath.itps.ncku.edu.tw/rnaseq/enrichment/Arabidopsis/enrichment_analysis.php, accessed on 25 August 2020) [95], and the enrichment terms were visualized. GO terms and KEGG pathways with FDR (false discovery rate) values of <0.05 and a *p*-value of <0.05, respectively, were considered.

## 5. Conclusions

Using RNA-Seq, we identified miRNAs in the WT Col-0 of *Arabidopsis* and its *abi1td*, *mkkk17*, and *mkkk18* mutant lines, which are differentially expressed in response to ABA treatment. Both known and novel miRNAs were characterized and the expression profiles of a subset were validated by qRT-PCR; we also verified the potential target genes of selected novel miRNAs by 5′ RLM-RACE. A hypothetical model of the ABA-responsive miRNA regulatory network and their target genes in *Arabidopsis* was developed from the data and is presented schematically (Figure 11). Overall, this study provides a database for the expansion of our knowledge and understanding of regulatory networks in the ABA core signaling pathway. These results will facilitate a broad understanding of the ABA response and help to clarify the miRNA-mediated molecular mechanisms of the fundamental response to ABA and provide insight into the functional role of miRNAs and their targets in *Arabidopsis* plants. Few of the novel *Arabidopsis* miRNAs detected in our study have previously been connected with the ABA response, especially in the WT Col-0 genotype. Therefore, it will be interesting to investigate the role of the ABA-responsive miRNAs that we report here, together with the proteins encoded by mRNAs targeted by these miRNAs, in plants responding to environmental challenges.

## Figures and Tables

**Table 1:** Mapping of small RNA-seq reads to the *Arabidopsis thaliana* genome of all libraries of *Arabidopsis* WT Col-0, *abi1td*, *mkkk17*, and *mkkk18* mutants before (mock) or after four hours of 100 µM ABA treatment (mock- and ABA-treated) in each of three biological replicates. Values with “±” in “[]” show standard deviation.

	Genotype and Condition	WT Col-0 Mock	WT Col-0 ABA	*abi1td* Mock	*abi1td* ABA	*mkkk17* Mock	*mkkk17* ABA	*mkkk18* Mock	*mkkk18* ABA
Unannotated	Processed Reads	9,484,333 (±305,722)	7,782,658 (±3,032,385)	10,327,250 (±1,263,563)	7,488,293 (±305,722)	12,597,403 (±306,139)	7,375,998 (±801,769)	10,332,359 (±593,100)	8,095,522 (±2,410,235)
	Aligned Reads	9,377,237 (±291,693) (98.87%)	7,592,660 (±2,990,770) (97.44%)	10,160,442 (±1,251,305) (98.38%)	7,356,650 (±933,688) (98.27%)	12,361,791 (±306,825) (98.13%)	7,256,364 (±783,546) (98.19%)	9,977,310 (±531,749) (96.58%)	7,837,873 (±2,319,507) (96.5%)
	alignment_not_unique	6,052,568 (±97,495)	4,860,241 (±2,226,793)	6,670,315 (±1,163,741)	4,657,135 (±640,197)	8,888,020 (±692,945)	4,130,142 (±523,786)	6,089,703 (±284,811)	4,864,610 (±1,607,750)
	ambiguous	84,483 (±28,631)	157,990 (±91,477)	205,679 (±51,593)	168,844 (±107,752)	242,764 (±65,097)	140,124 (±30,453)	252,149 (±25,429)	124,278 (±90,401)
	no_feature	1,095,681 (±320,538)	1,176,768 (±373,427)	1,386,570 (±112,773)	1,013,711 (±335,090)	1,439,914 (±210,521)	1,326,686 (±171,840)	1,781,633 (±301,685)	1,153,640 (±495,774)
	not_aligned	1,234,288 (±309,658)	589,360 (±116,084)	808,774 (±151,073)	651,428 (±363,168)	634,204 (±356,362)	731,196 (±258,725)	759,329 (±144,704)	1,010,286 (±153,079)
Annotated	lncRNA	15,888 (±4458)	17,414 (±4273)	19,856 (±2187)	15,312 (±4441)	19,546 (±3844)	18,929 (±3166)	24,911 (±4465)	16,666 (±6589)
	miRNA	360,701 (±94,021)	143,893 (±30,443)	327,809 (±70,037)	189,858 (±67,813)	246,958 (±126,301)	311,619 (±64,928)	391,059 (±79,742)	248,964 (±57,159)
	ncRNA	63,272 (±20,395)	16,918 (±7013)	53,668 (±7655)	31,245 (±17,723)	45,512 (±24,622)	54,202 (±6738)	71,275 (±14,488)	38,867 (±12,868)
	nontranslating_CDS	403 (±92)	431 (±163)	497 (±62)	395 (±122)	617 (±59)	400 (±26)	604 (±96)	401 (±196)
	protein_coding	282,368 (±32,142)	316,490 (±142,682)	341,844 (±30,871)	242,310 (±44,467)	484,528 (±36,738)	257,798 (±31,391)	354,631 (±58,945)	271,948 (±109,900)
	rRNA	103,274 (±8762)	131,380 (±33,382)	118,960 (±11,677)	106,636 (±22,877)	156,854 (±22,778)	102,371 (±21,861)	110,539 (±2074)	103,332 (±24,713)
	snRNA	2309 (±540)	2771 (±1457)	2922 (±272)	2168 (±517)	3696 (±762)	2619 (±180)	7647 (±6067)	2469 (±917)
	snoRNA	34,739 (±11,459)	37,995 (±19,310)	59,513 (±12,832)	54,093 (±24,850)	52,302 (±12,099)	27,799 (±4818)	56,471 (±17,001)	25,981 (±20,829)
	tRNA	154,356 (±24,306)	331,003 (±226,926)	330,838 (±67,629)	355,153 (±211,607)	382,484 (±123,782)	272,109 (±91,642)	432,406 (±108,854)	234,075 (±163,704)

**Table 2:** Normalized reads of top fifteen expressed miRNAs and their fold change in all genotypes. List of normalized reads of top fifteen expressed miRNAs in mock treatment and 4 h of 100 µM ABA-induced samples of *Arabidopsis thaliana* WT Col-0, *abi1td*, *mkkk17*, and *mkkk18* mutants in all libraries; each value is the average of three biological replicates of mock treatment and 4 h of 100 µM ABA-induced libraries. Fold change is from log2 (fold change) in NGS data after 4 h of 100 µM ABA-induced samples of *Arabidopsis thaliana* WT Col-0, *abi1td*, *mkkk17*, and *mkkk18* mutants; the *p*-value in NGS data is indicated with * (*p*-value ≤ 0.05), ** (*p*-value ≤ 0.01). ^†^ Values are in reads per million (RPM).

	Number of Reads ^†^								Fold Change (FC)			
miRNA ID	WT Col-0 Mock	WT Col-0 ABA	*abi1td* Mock	*abi1td* ABA	*mkkk17* Mock	*mkkk17* ABA	*mkkk18* Mock	*mkkk18 ABA*	WT Col-0	*abi1td*	*mkkk17*	*mkkk18*
miR166e-3p	20,943 (±5369)	24,743 (±7424)	16,015 (±4897)	16,298 (±511)	11,265 (±3877)	23,781 (±4607)	20,115 (±4559)	25,951 (±13,351)	0.56 *	0.26	−0.12	0.59
miR165b	5687 (±1575)	6133 (±1725)	4164 (±1477)	3676 (±159)	2601 (±942)	6084 (±1578)	5118 (±1218)	6266 (±4403)	0.43	0.07	0.02	0.51
miR159a	6732 (±1309)	4025 (±978)	4846 (±1625)	4444 (±1783)	2473 (±1437)	7751 (±2094)	5157 (±1625)	5706 (±1375)	−0.41	0.01	0.56	0.39
miR158a-3p	7803 (±2455)	1474 (±384)	6887 (±2683)	4128 (±2559)	4000 (±2460)	7378 (±2977)	6757 (±1703)	4582 (±712)	−1.99 **	−0.66	−0.18	−0.33
miR159b-3p	2886 (±584)	1468 (±320)	2072 (±856)	1357 (±463)	986 (±630)	2807 (±713)	2193 (±750)	1930 (±344)	−0.64 **	−0.45	0.46	0.06
miR398b-3p	1551 (±125)	1327 (±367)	786 (±368)	818 (±109)	473 (±208)	1202 (±455)	906 (±205)	1459 (±608)	0.09	0.29	0.16	0.91
miR167a-3p	1458 (±500)	1025 (±406)	1736 (±523)	1058 (±123)	1475 (±398)	1331 (±499)	3001 (±318)	1097 (±21)	−0.15	−0.48	−1.32 **	−1.24 **
miR160c-5p	448 (±125)	558.23 (±97)	351 (±138)	334 (±44)	215 (±91)	592 (±58)	463 (±72)	467.57 (±181)	0.69 **	0.16	0.29	0.22
miR827	1381 (±228)	597 (±139)	791 (±204)	897 (±250)	643 (±418)	1917 (±436)	1617 (±560)	1044 (±406)	−0.88 **	0.32	0.54	−0.38
miR403-3p	1120 (±317)	293 (±89)	1133 (±206)	926 (±588)	760 (±337)	1721 (±381)	1822 (±256)	1162 (±394)	−1.48 **	−0.27	0.03	−0.44
miR396a-5p	700 (±149)	317 (±34)	480 (±134)	462 (±260)	345 (±223)	861 (±115)	762 (±96)	570 (±95)	−0.75 **	0	0.28	−0.2
miR161.1	455 (±117)	396 (±99)	332 (±79)	356 (±52)	263 (±112)	532 (±170)	494 (±110)	492 (±157)	0.15	0.29	−0.15	0.22
miR167a-5p	401 (±103)	331 (±67)	382 (±105)	356 (±99)	246 (±128)	535 (±51)	487 (±81)	431 (±93)	0.09	0.04	0	0.05
miR156f-5p	363 (±60)	248 (±23)	291 (±128)	249 (±154)	152 (±73)	417 (±132)	367 (±65)	365 (±39)	−0.19	−0.13	0.23	0.18
miR408-3p	261 (±15)	427 (±202)	207 (±202)	288 (±66)	142 (±70)	467 (±82)	214 (±39)	404 (±39)	0.98 **	0.75 *	0.56 *	1.13 **

**Table 3:** Functional annotation of predicted target genes of differentially expressed novel miRNAs.

miRNA ID	Target Gene ID	Expectation	Inhibition	Target Description
ath-miRn-1	AT1G73390.3	2.5	Cleavage	Endosomal targeting BRO1-like domain-containing protein
ath-miRn-1	AT1G73390.4	2.5	Cleavage	Endosomal targeting BRO1-like domain-containing protein
ath-miRn-2	AT5G40550.1	3	Cleavage	SGF29 tudor-like domain
ath-miRn-4	AT3G14070.1	3.5	Cleavage	CAX9, CCX3, ATCCX3, cation exchanger 9
ath-miRn-6	AT1G56650.1	3	Cleavage	PAP1, MYB75, SIAA1, ATMYB75, production of anthocyanin pigment 1
ath-miRn-6	AT5G58490.1	3	Cleavage	NAD(P)-binding Rossmann-fold superfamily protein
ath-miRn-8	AT3G15570.1	3	Cleavage	Phototropic-responsive NPH3 family protein

## Data Availability

The datasets generated for this study are available on request to the corresponding author. The raw sequencing data (20 GB) were deposited at NCBI GEO with the accession number GSE172377.

## Supplementary Materials

The following are available online at https://www.mdpi.com/article/10.3390/ijms22137153/s1.
