# Supplementary material for: Identification of Novel miRNAs and Their Target Genes in the Response to Abscisic Acid in Arabidopsis
Source: Int J Mol Sci. 2021 Jul 1;22(13):7153. doi: 10.3390/ijms22137153 (PMC8268864; doi:10.3390/ijms22137153)

Supplementary Figures

Supplementary Figure S1: Clustering of sample correlations between *Arabidopsis* WT Col-0 , *abi1td, mkkk17 and mkkk18* mutants. The correlations of global expression between all samples were clustered and showing in a heatmap figure of *Arabidopsis* WT Col-0, *abi1td, mkkk17 and mkkk18* mutants with Mock and four hours ±100 μM ABA treated.

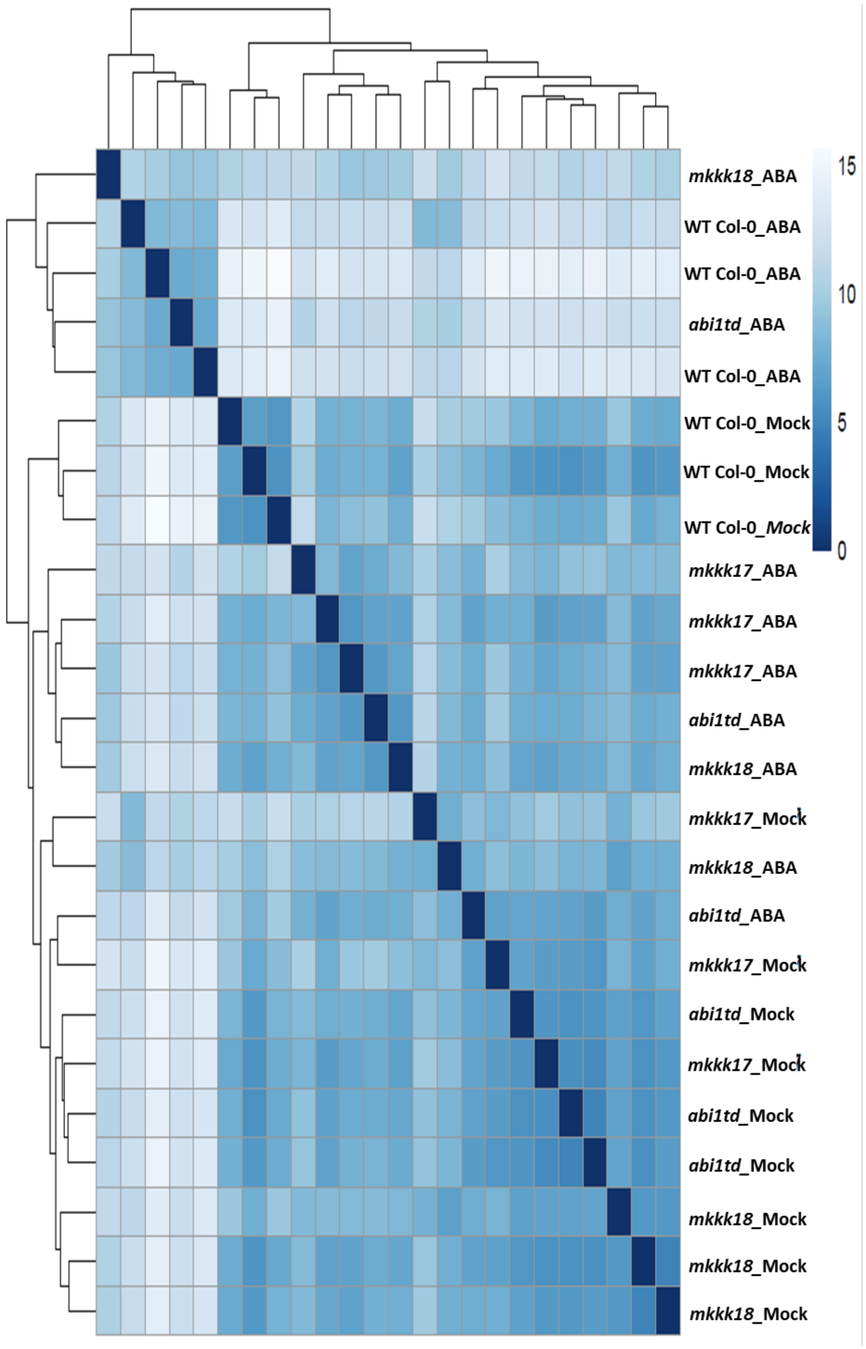

Supplementary Figure S2:

Secondary structures for the novel miRNA precursors. Predicted hairpin secondary structures for the potential novel miRNA precursors identified from small RNA deep sequencing (A–C) Predicted hairpin secondary structures for the potential novel miRNA precursors of (A) miRn-2 (B) miRn-6 (C) miRn-36. Nucleotide bases of mature miRNA are highlighted in light blue. The actual size of each putative precursor might differ slightly from its shown length since it was not identified experimentally. The computed minimum free energy (*MFE*) of the thermodynamic ensemble is reported. RNAstructure, Version 6.2 software was employed to evaluate the stem-loop structure with default parameter settings.

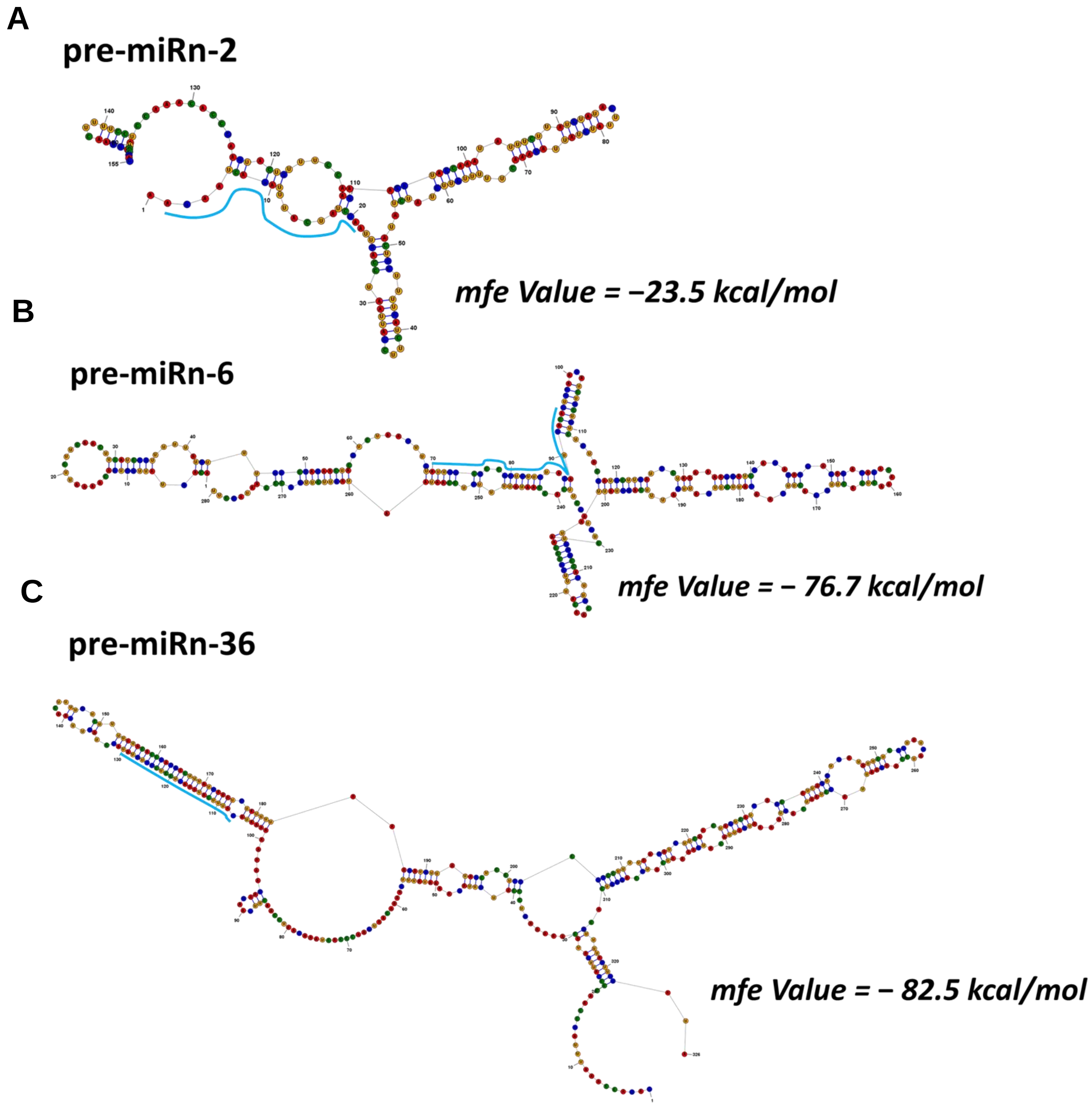

Supplement: Supplementary file 1 [file ijms-22-07153-s001.zip › Supplementary Figures.pdf]
